# Detection of H5N1 highly pathogenic avian influenza virus RNA in filth flies collected from California farms in 2024

**DOI:** 10.1038/s41598-025-29856-9

**Published:** 2025-11-28

**Authors:** Dana Nayduch, Stacey L.P. Scroggs, Phillip Shults, Luke A. Brendel, Lindsey M. Reister-Hendricks, Caitlin Taylor, Edward Bird, Brina Lopez, Edith S. Marshall

**Affiliations:** 1 Arthropod-Borne Animal Diseases Research Unit, Agricultural Research Service, Department of Agriculture, Manhattan, 66502 KS USA; 2 Department of Entomology, Kansas State University, Manhattan, KS 66506 USA; 3 California Department of Food and Agriculture, 1220 N Street, Sacramento, CA 95814 USA

**Keywords:** HPAI, Avian influenza, Flies, Dairy cattle, H5N1, *Musca domestica*, Microbiology, Molecular biology

## Abstract

The emergence of highly pathogenic avian influenza (HPAI) H5N1 clade 2.3.4.4b in U.S. dairy cattle highlights the urgent need to understand transmission dynamics within and among farms. House flies (*Musca domestica*) and blow flies (Calliphoridae), ubiquitous in agricultural settings, are suspected mechanical vectors of numerous pathogens, including viruses. We investigated the presence of H5N1 viral RNA in filth flies collected opportunistically from four H5N1-positive farms (three dairy and one poultry) in California during the 2024 outbreak. H5N1 RNA was detected via qRT-PCR in fly pools from all four locations, with the lowest Ct values (highest viral RNA) in house flies collected near milking parlors and dead animals. Whole-genome sequencing confirmed H5N1 viral genomes in flies from dairy farms, demonstrating high similarity (99.92–99.95%) to the B3.13 lineage circulating in the region and grouping closely with farm-associated viral sequences from milk. Although infectious virus was not detected in this study, our findings suggest that filth flies acquire HPAI H5N1 RNA from their environment, supporting their potential role as sentinels and/or mechanical vectors. These results underscore the critical importance of fly control, targeted surveillance, and integrated pest management strategies in agricultural settings to enhance biosecurity and potentially mitigate HPAI H5N1 transmission.

**Supplementary Information:**

The online version contains supplementary material available at 10.1038/s41598-025-29856-9.

## Introduction

Highly pathogenic avian influenza (HPAI) viruses are a threat to poultry worldwide, and spillover of HPAI H5N1 now poses a significant threat to mammals^1^. An ongoing outbreak of H5N1 clade 2.3.4.4b was first detected in avian, and later mammalian, species in North America in 2021^2–5^. Of particular concern was the detection of the virus in lactating dairy cattle in the U.S. in March 2024^6–8^. Lactating dairy cattle infected with this strain (clade 2.3.4.4b, genotype B3.13) demonstrated lethargy, decreased appetite and rumination, respiratory signs, milk production loss, and milk that is irregular in color and consistency^8,9^. The virus also spread to humans, mostly dairy farm employees, and caused fatal infections in domestic cats who drank unpasteurized milk^6,9,10^. The mechanism of transmission among dairy cattle is under debate with contaminated milking equipment, animal movement, and shared personnel all being investigated as possible transmission routes^7^, but there are other mechanisms to consider including insect-mediated dispersal and transmission.

House flies flourish in dairy cattle operations, where they have abundant access to breeding substrates (manure, spent feed) and food (animal waste, animal excretions, milk, feed, etc.). House flies and other filth flies have been well demonstrated to harbor, disperse, and/or transmit hundreds of species of microbial pathogens^11–14^. Adult house flies readily feed upon cattle excrement, wounds, and secretions such as mucus, tears and milk – particularly female flies who seek protein for vitellogenesis and oviposition^12,15^. Other filth flies, like Calliphorid blow flies, also infest livestock and poultry facilities, often in search of food and oviposition on dead animals or other decaying materials^16,17^. HPAI is not considered a classic arthropod-borne virus (arbovirus), but several fly species, including house flies, have been implicated as mechanical vectors of avian influenza viruses, including H5N1^18–25^. HPAI H5N1 remains infectious within the house fly body for up to 72 h and 24 h on the exterior fly surface, but viral copies slowly declined and did not replicate in the fly^18^. Additionally, chickens orally exposed to homogenized H5N1-infected flies became infected and died within 7 days^19^. HPAI H5N1 also has been detected in blow flies during outbreaks in poultry in Japan^22,25^.

Understanding how the HPAI H5N1 is spreading among animals such as dairy cattle within a farm and between farms is critical for outbreak management and biosecurity. The objective of this study was to determine if house flies and other filth flies carried HPAI H5N1 viral RNA and, therefore, potentially could be involved as sources of virus for later dispersal and/or as mechanical vectors for HPAI H5N1 in livestock operations.

## Results

### H5N1 RNA was detected in fly pools via qRT-PCR

All flies collected from dairies A, B and C were house flies (*M. domestica* L.) while flies collected from the poultry facility were all unspecified Calliphoridae (species undeterminable in the homogenized sample). H5N1 HPAI RNA was detected in flies sampled from all four locations (Table 1). Prevalence of viral RNA was greatest (indicated by lowest mean Ct values) in house flies collected near milking parlors or dead cattle (i.e., aborted fetus, carcass at Dairies A and B, respectively). Metadata for Dairy C were not available, so the locations within the facility from which flies were collected are unknown. Blow flies (Family: Calliphoridae) from the poultry facility were collected after a depopulation event. Of note, many additional fly pools also demonstrated qRT-PCR amplification of the target gene in multiple replicates but did not meet the cutoff of Ct value ≤ 36 and, therefore, were not classified as positive in this study. This included 10 samples from Dairy A (three from calf pail, seven from feed trough), 10 from Dairy B (eight from feed lane, two from carcass), 22 from Dairy C and 14 from the Poultry A location.

**Table 1:** qRT-PCR detection of H5N1 highly pathogenic avian influenza virus RNA in flies from four positive California farms.

Premise (county)^a^	% Pools Positive^b^ (*n*)	Average Ct^c^ (SE)	Fly type
Dairy A (Fresno)			
Aborted Fetus	100.0 (20)	28.98 (0.42)	House
Calf Feed Pail	0.0 (30)	N/A	House
Feed Trough	10.0 (20)	34.06 (0.05)	House
Parlor Dumpster	100.0 (10)	27.13 (0.68)	House
Dairy B (Kings)			
Feed Lane	12.5 (40)	33.00 (2.42)	House
Parlor	96.7 (30)	32.17 (0.27)	House
Carcass	60.0 (10)	34.00 (0.55)	House
Poultry A (Tulare)	10.0 (100)	34.00 (0.52)	Blow
Dairy C (Merced)	59.0 (100)	32.70 (0.28)	House

^a^Collection dates were: Dairy A, 10/12/2024; Dairy B, 10/21/2024; Poultry A, 11/6/2024; Dairy C, 11/13/2024.

^b^Positive is Ct value ≤ 36 and *n* is total number of pools (5 flies per pool).

^c^Ct = Threshold cycle in qRT-PCR.

### Whole genome H5N1 sequences were generated from fly pools

**Fig. 1:**
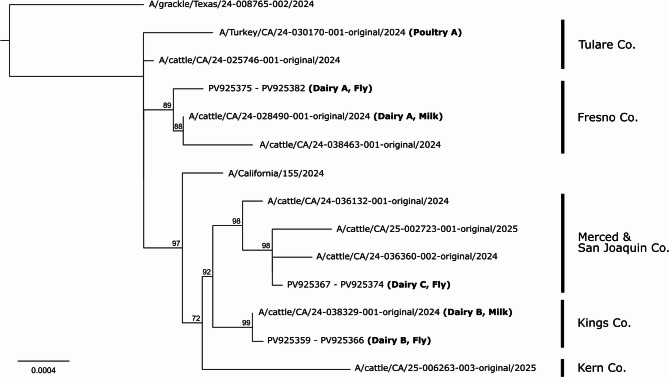
Maximum likelihood tree of whole genome viral sequences obtained from fly samples in this study (PV925359 - PV925359) as well as closely related publicly available sequences (labeled as A/host/state/sample_ID/collection year; see BioProject: PRJNA1102327, https://www.biorxiv.org/content/10.1101/2024.05.01.591751v1). Viruses originating from premises included in this study as well as their source of origin are bolded in parentheses. Bootstrap values greater than 50 are shown next to the corresponding node. When available, the county of origin for each sequence is indicated to the right. The width of the scale bar indicates 0.0004 expected substitutions per site (averaged across partitions). Branch lengths are proportional to the estimated number of substitutions per site.

While RT amplification was observed in individual fly pools collected from each of the four sites (Dairy A, B, C and Poultry A), no influenza reads were recovered from the blow flies collected at Poultry A. The remaining results are from house flies collected from the three dairies. After filtering, the number of raw reads per sample was ~ 750,000–5,000,000; however, less than 1% of these reads mapped to the H5N1 genome due to co-amplification of fly and other contaminant RNA during RT-PCR. There was a wide range in genome coverage between house fly samples; the coverage from Dairy A was ~ 2,162×, Dairy B was 5×, and Dairy C was 26×. Still, whole H5N1 viral genomes were obtained from flies at Dairy A and C, with a partial genome obtained from Dairy B (92% complete). Samples from dairy A and C were determined to be of genotype B3.13 (PB1:am4, PB2:am2.2, PA: aa1, HA: ea1, NP: am8, NA: ea1, MP: ea1, NS: am1.1) by GenoFlu. Due to the incomplete sequencing of the HA gene segment for the sample from dairy B, GenoFlu was unable to assign a genotype to this sample. HA was classified as ea2, which has not been previously documented as reassorting into B3 genotypes. However, when compared to sequences presented in this study, the HA region from dairy B had 0–2 mismatches with all other samples included in this study. These genomes were found have an overall sequence similarity of 99.92–99.95% and belonged to the B3.13 lineage of H5N1. Viral genomic sequences from the house flies were extremely closely related to sequences from the milk or tracheal swab samples collected from the same premises (99.97–99.99%). Phylogenetic analyses of these sequences also showed county-level clustering within the clades on the tree (Fig. 1).

## Discussion

This study, undertaken opportunistically during a massive outbreak emergency response, represents the first account of filth flies carrying HPAI H5N1 RNA at farming operations (three dairy, one poultry) during the 2024 outbreak in California. The role of flies, particularly house flies (*Musca domestica*) and blowflies (e.g., *Calliphora nigribarbis*), as potential mechanical vectors for the spread of the H5N1 avian influenza virus previously has been considered, both by detecting infectious virus or viral RNA in field surveys and by demonstrating virus survival in flies via feeding assays^18,24^. Prior bioassays determined that infectious HPAI H5N1 survives in the digestive tract (including crop) of house flies for up to 72 h^18^ and blow flies for up to 24 h^24^, although viral RNA can be detected for much longer (for example, up to 4 days in house flies and 14 days in blow flies). Low pathogenic avian influenza virus (H9N2) similarly survived in house flies up to 24 h on the body surface and up to 96 h internally, as determined via feeding assays^26^. Thus, it is important to note that only viral RNA was detected from flies in the current study, and live/infectious virus must be detected in order to conclusively incriminate filth flies as sources, dispersal agents and/or transmitters of infectious HPAI H5N1 during the current 2024–2025 outbreak. Nonetheless, because viral RNA was readily detected both by qRT-PCR and sequencing, house flies may serve as sentinels for monitoring the presence of HPAI H5N1 in addition to bulk tank sampling of milk, which is the main mode of surveillance at dairy farms.

The successful amplification and sequencing of full-length HPAI H5N1 genomic DNA from fly samples collected from positive dairy operations, along with the incidence of relatively low Ct values in some pools, together suggest the presence of intact viral genomes at the time of fly collection. However, the detection of intact genomes does not necessarily equate to the presence of infectious virus, which is a limitation of our approach that could be improved if virus culture precedes molecular characterization. Furthermore, because HPAI H5N1 RNA was detected from homogenized pools of flies, the precise location of the virus—whether on the external surface and/or within the alimentary canal—remains to be determined. Delineating this location is critical for assessing vectoring potential as microbes present on the external surface of flies are more susceptible to desiccation^27^, whereas ingested microbes, particularly those temporarily stored in the crop, may accumulate and stay protected in consumed substrates and potentially can be transmitted through subsequent regurgitation^14^.

Our data suggest that both the milking parlor and dead animals may be potentially potent sources of virus for flies on the two dairy operations for which we had these metadata, as indicated by low Ct values (i.e., high viral RNA template), a high prevalence of positive fly pools collected from these locations, and the close relatedness of genomes of fly-associated and milk-associated virus from the same farm and nearby farms in the same county (e.g., Fig. 1). Together, these findings support local acquisition of HPAI H5N1 by house flies from both milk and dead animals (carcasses, aborted fetus), which concurs with the trophic behaviors of filth flies. Female filth flies readily seek out and feed on proteinaceous substances like milk (e.g., house flies;^15^ and both species visit dead or decaying materials for oviposition^28,29^, often ingesting the substrate during this process. Interestingly, milk seems to extend the stability of HPAI H5N1 in wastewater and on structural surfaces^30,31^. Milk also has been shown to increase persistence, perhaps by decreasing degradation rates, of SARS-CoV-2 ingested by house flies^32^.

House flies and blow flies tend to stay near rich sources of food and reproductive substrates, but will disperse in search of new sources when these become limited^33,34^. Activities such as the removal (including depopulation) of animals, their waste and their feed could drive fly dispersal. Movement of house flies has been estimated to be over 1.6 km^35^ when resources are present, for example between breeding and feeding sites, but dispersal over 12 km has been reported in several studies^36–38^. Blow flies also are motivated by the availability of resources, but some species can disperse up to 45 km, although the transit time for this long dispersal is not known^39^. These estimates for either fly species neither take into account the abovementioned triggers for dispersal, such as food or reproductive site depletion, nor confounding environmental factors that may limit or promote dispersal.

Our ongoing surveys of flies for HPAI H5N1 are focused on estimating the incidence and prevalence of both viral RNA and infectious virus in individual house flies. This research aims to better understand and subsequently model the potential for viral acquisition, dispersal, and transmission by these livestock pests. The implications of our findings, and the aim of our ongoing work, is to determine whether controlling fly populations, particularly in agricultural settings, may be instrumental in promoting biosecurity and, potentially, managing infectious disease outbreaks including HPAI H5N1. While detection of live virus in flies from infected farms remains to be determined, the evidence presented in our study strongly supports the necessity for further research into the role these mechanical vectors play in outbreaks of HPAI in dairy and poultry facilities. Nonetheless, because viral RNA was easily detected and highly prevalent in flies during an active outbreak, they stand to, at the very least, serve as surveillance tools for this important virus.

## Methods

### Collection sites and fly collections

A convenience sample of farms was voluntarily enrolled in this fly sampling study in conjunction with their voluntary participation in sampling of wildlife and peri-domestic vertebrates during California’s unprecedented H5N1 outbreak of 2024-25. Potential sites were identified through both active and passive surveillance, and information about potential research study participation was provided after detection through emergency response staff and outreach. All sites were confirmed positive for HPAI virus clade 2.3.4.4b, genotype B3.13, prior to selection via bulk tank milk sampling or animal samples (e.g., tracheal swabs from poultry carcasses). Each participating site was located in the central valley of California with Dairy A in Fresno County (herd size > 9,000), Dairy B in Kings County (unreported herd size), Dairy C in Merced County (herd size < 3,000), and Poultry A in Tulare County (~ 750,000 birds). The presumptive positive dates for all locations were between late September to late October 2024.

Fly collection was performed by field scientists from the USDA’s Animal and Plant Health Inspection Service (APHIS) – Wildlife Services (WS) in conjunction with the concurrent vertebrate surveys being performed on these H5N1-positive premises. Collection was performed within 3 weeks of initial viral detection at each site. Flies were collected by sweep netting then pooled by species, with the goal being to collect filth flies, i.e., house flies (*Musca domestica* L.) or blowflies (Family: Calliphoridae). Pools of 5 individuals were placed in 500 µl of DNA/RNA Shield (Zymo Research, Irvine, CA, USA) and briefly homogenized with a sterile pestle to release contents, neutralize pathogens and preserve nucleic acids. Descriptive metadata, such as site within the farm, were noted for samples (e.g., milking parlor, carcass pile) when available. Samples were shipped overnight to the USDA’s Arthropod-Borne Animal Diseases Research Unit in Manhattan, KS, USA.

### Viral RNA extraction and RT-qPCR for H5N1 detection

Fly pools were further homogenized by hand with a pestle briefly before the addition of one 5/32” stainless steel bead per tube. The samples were homogenized using the Bead Ruptor Elite (Omni International, Kennesaw, GA, USA) for 30 seconds at 0.2m/s and clarified via centrifugation at 21,000 g for one minute to pellet tissue debris. RNA was extracted from 200 µl clarified homogenate using the MagMAX CORE Nucleic Acid Purification Kit and the KingFisher X Apex System (Applied Biosystems, Thermo Fisher Scientific, Inc. Waltham, MA, USA)^40^. Extracted RNA was stored at -80 °C. Positive H5N1 RNA was extracted from 38-ADV inactivated Influenza A Virus rg-A/bald eagle/FL/W22-134-OP/2022 (H5N1), kindly provided by the USDA-APHIS National Veterinary Services Laboratory using the same methods. H5N1 RNA was detected by the 7500 Fast PCR Detection System (Applied Biosystems, Thermo Fisher Scientific, Inc. Waltham, MA, USA) with the AgPath ID RT-PCR kit (Applied Biosystems, Thermo Fisher Scientific, Inc. Waltham, MA, USA) which targets the Matrix gene using the following primers F25 forward: 5’ AGATGAGTCTTCTAACCGAGGTCG 3’, R124 reverse: 5’TGCAAAAACATCTTCAAGTCTCTG3’, R124-modified reverse primer: 5’TGCAAAGACACTTTCCAGTCTCTG3’, and F64 probe: FAM-TCAGGCCCCCTCAAAGCCGA-BHQ-1^41^. The following amplification conditions were used: Reverse transcription was completed with one cycle of 10 min at 45 °C and 10 min at 95 °C, followed by amplification using 45 cycles of 15 s at 95 °C and 45 s at 60 °C^41^. All PCR reactions were conducted in triplicate and included a negative water control and positive H5N1 control. Samples with a threshold cycle (Ct) value ≤ 36 in at least two of three replicates were considered positive and were subsequently confirmed as RNA from strain H5N1 by sequencing as described below.

### H5N1 genome sequencing and analysis

Extracted RNA from H5N1-positive pools were subject to whole-genome sequencing. Whole-genome amplification of H5N1 was performed on one random sample from each site using a SuperScript IV One-Step RT-PCR kit (Invitrogen, Thermo Fisher Scientific, Inc. Waltham, MA, USA) and the Uni/Inf primer set^42^. For each sample, 2.5 ul of RNA was added to 12.5 µl of RT-PCR Master Mix, 0.5 µl of SuperScript™ IV RT Mix, 7.0 µl of water, 0.5 µl of Uni-12/Inf1, 0.75 µl of Uni-12/Inf3, and 1.25 µl of Uni-13/Inf1 (all primers were at 10 µM). The samples were then run on a Bio-Rad T100 Thermal Cycler (Bio-Rad, Hurcules, CA, USA). The thermal cycling conditions began with a hot start RT step at 55 °C for 2 min, followed by 42 °C for 90 min and 98 °C for 2 min. Following this were 5 cycles of 98 °C for 10 s, 45 °C for 30 s, and 72 °C for 3 min 30 s; then a second set of cycling for 35 cycles at 98 °C for 10 s, 67 °C for 30 s, and 72 °C for 3 min 30 s, with a final extension of 72 °C for 10 min^43^. PCR products were visualized using gel electrophoresis and purified with AMPure XP beads (Beckman Coulter, Brea, CA, USA) at a ratio of 0.5x. Libraries were generated from 500 ng of cDNA using an Elevate Enzymatic Library Prep kit (Element Biosciences, San Diego, CA, USA) targeting a 250–350 bp insert size. Sequencing was performed on an AVITI platform (Element Biosciences, San Diego, CA, USA) in a 75 bp paired-end run.

The resulting paired-end sequencing data were trimmed and filtered using the ‘element_cleanup’ pipeline (https://github.com/edwardbirdlab/element_cleanup) to remove Element specific sequencing artifacts. This pipeline employed BBDuk v38.96 and fastp v0.24.0 to correct sequencing errors associated with avidity-based chemistry^44,45^. Consensus sequences were subsequently generated from the filtered paired-end data using the nf-core viralrecon pipeline (https://nf-co.re/viralrecon/2.6.0/) in metagenomic mode, as sequencing primers were absent from the provided reference (GCA_049137395.1). Viralrecon v2.6.0 also used fastp v0.23.4 for quality trimming, Bowtie2 v2.4.4 and SAMtools v1.16.1 for alignment to the supplied HPAI reference genome, and BCFtools v1.16 to generate consensus sequences^46–49^. The exact commands used for the bioinformatics workflow are available in a public GitHub repository (https://github.com/edwardbirdlab/hapi_methods2025). Consensus sequences (*n* = 3) were genotyped utilizing GenoFlu v1.06, and supplementary alignments were performed using the Biopython PairwiseAligner v1.86 with the Needleman-Wunsch algorithm, with settings of match score = 2, mismatch score = -1, open gap score = -2 and extend gap score = -0.5. Sequences obtained in this study (*n* = 3), as well as publicly available sequences of the B3.13 lineage of H5N1 (*n* = 11), were used in creating a phylogenetic tree. The publicly available sequences were selected based on time of collection and locality. Each of the 8 genome segments was aligned separately using the Geneious alignment tool using the default setting in Geneious Prime v2025.0.3^50^ and then combined for a total of 13,136 bp. A partition file denoting each segment was created for use in constructing the phylogenetic tree. A maximum likelihood tree was produced using the RAxML plugin for Geneious using a GTR GAMMA substitution model (determined to be the most appropriate for each partition using ModelFinder) with 100 rapid bootstrap replicates^51^.

## Supplementary Information

Below is the link to the electronic supplementary material.

Supplementary Material 1

## Data Availability

All data generated during this study are included in this published article and its Supplemental Information files. HPAI viral sequences were deposited into GenBank under accession numbers PV925359-PV925382.
