# Supplementary material for: Detection of H5N1 highly pathogenic avian influenza virus RNA in filth flies collected from California farms in 2024
Source: Sci Rep. 2025 Nov 28;16:375. doi: 10.1038/s41598-025-29856-9 (PMC12770564; doi:10.1038/s41598-025-29856-9)
Supplement: Supplementary file 1 — Supplementary Material 1 [file 41598_2025_29856_MOESM1_ESM.pdf]

Tube #	Site Name	Sample Origin	Avg Ct (triplicate)
1	Dairy A	Aborted Fetus	29.80761782
2	Dairy A	Aborted Fetus	30.79345957
3	Dairy A	Aborted Fetus	26.97185898
4	Dairy A	Aborted Fetus	29.61549886
5	Dairy A	Aborted Fetus	29.45282491
6	Dairy A	Aborted Fetus	26.20275879
7	Dairy A	Aborted Fetus	28.7703317
8	Dairy A	Aborted Fetus	25.59537125
9	Dairy A	Aborted Fetus	27.65695127
10	Dairy A	Aborted Fetus	29.09188143
11	Dairy A	Aborted Fetus	33.34246318
12	Dairy A	Aborted Fetus	27.05121168
13	Dairy A	Aborted Fetus	29.48996735
14	Dairy A	Aborted Fetus	29.93646113
15	Dairy A	Aborted Fetus	28.17741776
16	Dairy A	Aborted Fetus	30.95955404
17	Dairy A	Aborted Fetus	28.77985319
18	Dairy A	Aborted Fetus	30.9020799
19	Dairy A	Aborted Fetus	29.85187976
20	Dairy A	Aborted Fetus	27.04703522
21	Dairy A	Calf Feed Pail	Undetected
22	Dairy A	Calf Feed Pail	Undetected
23	Dairy A	Calf Feed Pail	Undetected
24	Dairy A	Calf Feed Pail	Undetected
25	Dairy A	Calf Feed Pail	Undetected
26	Dairy A	Calf Feed Pail	Undetected
27	Dairy A	Calf Feed Pail	Undetected
28	Dairy A	Calf Feed Pail	Undetected
29	Dairy A	Calf Feed Pail	Undetected
30	Dairy A	Calf Feed Pail	Undetected
31	Dairy A	Calf Feed Pail	Undetected
32	Dairy A	Calf Feed Pail	Undetected
33	Dairy A	Calf Feed Pail	Undetected
34	Dairy A	Calf Feed Pail	Undetected
35	Dairy A	Calf Feed Pail	Undetected
36	Dairy A	Calf Feed Pail	Undetected
37	Dairy A	Calf Feed Pail	Undetected
38	Dairy A	Calf Feed Pail	36.46310806
39	Dairy A	Calf Feed Pail	Undetected
40	Dairy A	Calf Feed Pail	Undetected
41	Dairy A	Calf Feed Pail	Undetected
42	Dairy A	Calf Feed Pail	Undetected
43	Dairy A	Calf Feed Pail	Undetected
44	Dairy A	Calf Feed Pail	37.08309364
45	Dairy A	Calf Feed Pail	Undetected
46	Dairy A	Calf Feed Pail	Undetected
47	Dairy A	Calf Feed Pail	Undetected

48	Dairy A	Calf Feed Pail	36.28825887
49	Dairy A	Calf Feed Pail	Undetected
50	Dairy A	Calf Feed Pail	Undetected
51	Dairy A	Fresh Cow Feed Trough	36.88135338
52	Dairy A	Fresh Cow Feed Trough	34.01288223
53	Dairy A	Fresh Cow Feed Trough	36.54783376
54	Dairy A	Fresh Cow Feed Trough	Undetected
55	Dairy A	Fresh Cow Feed Trough	36.38383102
56	Dairy A	Fresh Cow Feed Trough	Undetected
57	Dairy A	Fresh Cow Feed Trough	Undetected
58	Dairy A	Fresh Cow Feed Trough	Undetected
59	Dairy A	Fresh Cow Feed Trough	36.44964981
60	Dairy A	Fresh Cow Feed Trough	37.8408947
61	Dairy A	Fresh Cow Feed Trough	Undetected
62	Dairy A	Fresh Cow Feed Trough	36.32685343
63	Dairy A	Fresh Cow Feed Trough	Undetected
64	Dairy A	Fresh Cow Feed Trough	Undetected
65	Dairy A	Fresh Cow Feed Trough	36.47865105
66	Dairy A	Fresh Cow Feed Trough	Undetected
67	Dairy A	Fresh Cow Feed Trough	Undetected
68	Dairy A	Fresh Cow Feed Trough	37.42092323
69	Dairy A	Fresh Cow Feed Trough	Undetected
70	Dairy A	Fresh Cow Feed Trough	34.1029803
71	Dairy A	Dumpster Near Parlor	28.8850282
72	Dairy A	Dumpster Near Parlor	29.95320765
73	Dairy A	Dumpster Near Parlor	25.80287107
74	Dairy A	Dumpster Near Parlor	28.94627889
75	Dairy A	Dumpster Near Parlor	26.96200466
76	Dairy A	Dumpster Near Parlor	27.4447298
77	Dairy A	Dumpster Near Parlor	25.61917051
78	Dairy A	Dumpster Near Parlor	26.58639781
79	Dairy A	Dumpster Near Parlor	28.51158905
80	Dairy A	Dumpster Near Parlor	22.6027813
81	Dairy B	Feed Lanes	37.63479487
82	Dairy B	Feed Lanes	Undetected
83	Dairy B	Feed Lanes	Undetected
84	Dairy B	Feed Lanes	Undetected
85	Dairy B	Feed Lanes	Undetected
86	Dairy B	Feed Lanes	Undetected
87	Dairy B	Feed Lanes	Undetected
88	Dairy B	Feed Lanes	Undetected
89	Dairy B	Feed Lanes	Undetected
90	Dairy B	Feed Lanes	Undetected
91	Dairy B	Feed Lanes	Undetected
92	Dairy B	Feed Lanes	Undetected
93	Dairy B	Feed Lanes	37.99976603
94	Dairy B	Feed Lanes	Undetected
95	Dairy B	Feed Lanes	Undetected

96	Dairy B	Feed Lanes	Undetected
97	Dairy B	Feed Lanes	Undetected
98	Dairy B	Feed Lanes	Undetected
99	Dairy B	Feed Lanes	Undetected
100	Dairy B	Feed Lanes	Undetected
101	Dairy B	Feed Lanes	Undetected
102	Dairy B	Feed Lanes	Undetected
103	Dairy B	Feed Lanes	Undetected
104	Dairy B	Feed Lanes	Undetected
105	Dairy B	Feed Lanes	Undetected
106	Dairy B	Feed Lanes	35.43108114
107	Dairy B	Feed Lanes	Undetected
108	Dairy B	Feed Lanes	Undetected
109	Dairy B	Feed Lanes	Undetected
110	Dairy B	Feed Lanes	30.58825239
111	Dairy B	Feed Lanes	36.94404602
112	Dairy B	Feed Lanes	36.79389826
113	Dairy B	Feed Lanes	Undetected
114	Dairy B	Feed Lanes	37.79030991
115	Dairy B	Feed Lanes	35.6315829
116	Dairy B	Feed Lanes	37.12187386
117	Dairy B	Feed Lanes	35.60342534
118	Dairy B	Feed Lanes	37.91423798
119	Dairy B	Feed Lanes	35.90229797
120	Dairy B	Feed Lanes	38.50945663
121	Dairy B	In & Around Parlor	33.90225983
122	Dairy B	In & Around Parlor	35.35066605
123	Dairy B	In & Around Parlor	32.76012929
124	Dairy B	In & Around Parlor	34.6968956
125	Dairy B	In & Around Parlor	31.1580658
126	Dairy B	In & Around Parlor	32.1257782
127	Dairy B	In & Around Parlor	30.61192703
128	Dairy B	In & Around Parlor	31.92204666
129	Dairy B	In & Around Parlor	31.71115239
130	Dairy B	In & Around Parlor	33.19393158
131	Dairy B	In & Around Parlor	31.58293406
132	Dairy B	In & Around Parlor	43.46047974
133	Dairy B	In & Around Parlor	31.66178322
134	Dairy B	In & Around Parlor	32.09140714
135	Dairy B	In & Around Parlor	30.93676058
136	Dairy B	In & Around Parlor	30.8796463
137	Dairy B	In & Around Parlor	30.46666463
138	Dairy B	In & Around Parlor	32.55297915
139	Dairy B	In & Around Parlor	29.06364568
140	Dairy B	In & Around Parlor	32.45379066
141	Dairy B	In & Around Parlor	33.61949666
142	Dairy B	In & Around Parlor	33.22179921
143	Dairy B	In & Around Parlor	32.24385579

144	Dairy B	In & Around Parlor	32.71001816
145	Dairy B	In & Around Parlor	31.88211759
146	Dairy B	In & Around Parlor	33.60317866
147	Dairy B	In & Around Parlor	33.78444544
148	Dairy B	In & Around Parlor	32.3814888
149	Dairy B	In & Around Parlor	30.58076413
150	Dairy B	In & Around Parlor	29.65404956
151	Dairy B	Carcass Pile	33.84878159
152	Dairy B	Carcass Pile	35.22259267
153	Dairy B	Carcass Pile	33.59303284
154	Dairy B	Carcass Pile	31.5640475
155	Dairy B	Carcass Pile	Undetected
156	Dairy B	Carcass Pile	34.90482585
157	Dairy B	Carcass Pile	34.81637955
158	Dairy B	Carcass Pile	36.32148361
159	Dairy B	Carcass Pile	Undetected
160	Dairy B	Carcass Pile	36.14443207
161	Poultry A	n/a	Undetected
162	Poultry A	n/a	31.40736008
163	Poultry A	n/a	Undetected
164	Poultry A	n/a	Undetected
165	Poultry A	n/a	Undetected
166	Poultry A	n/a	Undetected
167	Poultry A	n/a	Undetected
168	Poultry A	n/a	Undetected
169	Poultry A	n/a	Undetected
170	Poultry A	n/a	37.31189728
171	Poultry A	n/a	Undetected
172	Poultry A	n/a	32.8632253
173	Poultry A	n/a	Undetected
174	Poultry A	n/a	37.83486938
175	Poultry A	n/a	Undetected
176	Poultry A	n/a	Undetected
177	Poultry A	n/a	Undetected
178	Poultry A	n/a	37.22909673
179	Poultry A	n/a	37.53904724
180	Poultry A	n/a	Undetected
181	Poultry A	n/a	Undetected
182	Poultry A	n/a	Undetected
183	Poultry A	n/a	33.16270828
184	Poultry A	n/a	Undetected
185	Poultry A	n/a	Undetected
186	Poultry A	n/a	35.4679985
187	Poultry A	n/a	Undetected
188	Poultry A	n/a	Undetected
189	Poultry A	n/a	Undetected
190	Poultry A	n/a	36.96731949
191	Poultry A	n/a	Undetected

192	Poultry A	n/a	Undetected
193	Poultry A	n/a	Undetected
194	Poultry A	n/a	Undetected
195	Poultry A	n/a	Undetected
196	Poultry A	n/a	Undetected
197	Poultry A	n/a	37.94327736
198	Poultry A	n/a	Undetected
199	Poultry A	n/a	Undetected
200	Poultry A	n/a	38.77578735
201	Poultry A	n/a	Undetected
202	Poultry A	n/a	34.78168233
203	Poultry A	n/a	Undetected
204	Poultry A	n/a	Undetected
205	Poultry A	n/a	37.94247818
206	Poultry A	n/a	Undetected
207	Poultry A	n/a	Undetected
208	Poultry A	n/a	Undetected
209	Poultry A	n/a	39.66272354
210	Poultry A	n/a	Undetected
211	Poultry A	n/a	Undetected
212	Poultry A	n/a	Undetected
213	Poultry A	n/a	36.85815048
214	Poultry A	n/a	39.43605995
215	Poultry A	n/a	Undetected
216	Poultry A	n/a	Undetected
217	Poultry A	n/a	38.74188614
218	Poultry A	n/a	Undetected
219	Poultry A	n/a	Undetected
220	Poultry A	n/a	35.81209564
221	Poultry A	n/a	Undetected
222	Poultry A	n/a	Undetected
223	Poultry A	n/a	Undetected
224	Poultry A	n/a	Undetected
225	Poultry A	n/a	Undetected
226	Poultry A	n/a	Undetected
227	Poultry A	n/a	Undetected
228	Poultry A	n/a	Undetected
229	Poultry A	n/a	Undetected
230	Poultry A	n/a	Undetected
231	Poultry A	n/a	Undetected
232	Poultry A	n/a	Undetected
233	Poultry A	n/a	Undetected
234	Poultry A	n/a	Undetected
235	Poultry A	n/a	Undetected
236	Poultry A	n/a	Undetected
237	Poultry A	n/a	Undetected
238	Poultry A	n/a	36.85055161
239	Poultry A	n/a	Undetected

240	Poultry A	n/a	Undetected
241	Poultry A	n/a	Undetected
242	Poultry A	n/a	Undetected
243	Poultry A	n/a	Undetected
244	Poultry A	n/a	Undetected
245	Poultry A	n/a	Undetected
246	Poultry A	n/a	Undetected
247	Poultry A	n/a	Undetected
248	Poultry A	n/a	31.47195053
249	Poultry A	n/a	34.7473774
250	Poultry A	n/a	35.36107635
251	Poultry A	n/a	Undetected
252	Poultry A	n/a	Undetected
253	Poultry A	n/a	Undetected
254	Poultry A	n/a	Undetected
255	Poultry A	n/a	Undetected
256	Poultry A	n/a	Undetected
257	Poultry A	n/a	Undetected
258	Poultry A	n/a	37.04500961
259	Poultry A	n/a	Undetected
260	Poultry A	n/a	35.10008621
261	Dairy C	n/a	33.99679438
262	Dairy C	n/a	33.79610316
263	Dairy C	n/a	Undetected
264	Dairy C	n/a	Undetected
265	Dairy C	n/a	34.34670512
266	Dairy C	n/a	Undetected
267	Dairy C	n/a	Undetected
268	Dairy C	n/a	Undetected
269	Dairy C	n/a	34.73871613
270	Dairy C	n/a	35.95470937
271	Dairy C	n/a	Undetected
272	Dairy C	n/a	31.47026253
273	Dairy C	n/a	32.81447728
274	Dairy C	n/a	32.80686251
275	Dairy C	n/a	31.60923576
276	Dairy C	n/a	35.07609558
277	Dairy C	n/a	30.48638916
278	Dairy C	n/a	Undetected
279	Dairy C	n/a	Undetected
280	Dairy C	n/a	30.03736051
281	Dairy C	n/a	33.44868088
282	Dairy C	n/a	30.94295247
283	Dairy C	n/a	25.50617727
284	Dairy C	n/a	30.6588974
285	Dairy C	n/a	32.90461477
286	Dairy C	n/a	31.83421961
287	Dairy C	n/a	31.29318047

288	Dairy C	n/a	29.27071444
289	Dairy C	n/a	30.77266312
290	Dairy C	n/a	29.93687185
291	Dairy C	n/a	35.99385071
292	Dairy C	n/a	34.34135056
293	Dairy C	n/a	33.8651104
294	Dairy C	n/a	30.74625524
295	Dairy C	n/a	30.16695404
296	Dairy C	n/a	31.75084305
297	Dairy C	n/a	34.20584869
298	Dairy C	n/a	31.27345085
299	Dairy C	n/a	32.34798622
300	Dairy C	n/a	32.8783989
301	Dairy C	n/a	30.84093539
302	Dairy C	n/a	31.3175621
303	Dairy C	n/a	31.41748556
304	Dairy C	n/a	29.59227053
305	Dairy C	n/a	36.44138082
306	Dairy C	n/a	35.1169459
307	Dairy C	n/a	40.9068222
308	Dairy C	n/a	38.39130402
309	Dairy C	n/a	38.62401835
310	Dairy C	n/a	40.17678833
311	Dairy C	n/a	39.69997787
312	Dairy C	n/a	37.7353948
313	Dairy C	n/a	38.1372261
314	Dairy C	n/a	38.48393504
315	Dairy C	n/a	32.85763931
316	Dairy C	n/a	35.37203725
317	Dairy C	n/a	37.75253677
318	Dairy C	n/a	38.19750722
319	Dairy C	n/a	36.02382278
320	Dairy C	n/a	37.57154846
321	Dairy C	n/a	39.63114293
322	Dairy C	n/a	Undetected
323	Dairy C	n/a	35.18827248
324	Dairy C	n/a	30.19254621
325	Dairy C	n/a	37.81144333
326	Dairy C	n/a	34.3965594
327	Dairy C	n/a	36.12382317
328	Dairy C	n/a	Undetected
329	Dairy C	n/a	37.40899467
330	Dairy C	n/a	Undetected
331	Dairy C	n/a	36.57965469
332	Dairy C	n/a	Undetected
333	Dairy C	n/a	Undetected
334	Dairy C	n/a	32.97225952
335	Dairy C	n/a	34.69003805

336	Dairy C	n/a	Undetected
337	Dairy C	n/a	35.26836777
338	Dairy C	n/a	30.06357956
339	Dairy C	n/a	35.45487213
340	Dairy C	n/a	33.31442897
341	Dairy C	n/a	Undetected
342	Dairy C	n/a	35.22770945
343	Dairy C	n/a	Undetected
344	Dairy C	n/a	30.43471082
345	Dairy C	n/a	30.29156621
346	Dairy C	n/a	34.9191494
347	Dairy C	n/a	33.14544551
348	Dairy C	n/a	36.63479233
349	Dairy C	n/a	34.5734272
350	Dairy C	n/a	35.22139359
351	Dairy C	n/a	32.56253115
352	Dairy C	n/a	Undetected
353	Dairy C	n/a	34.64876175
354	Dairy C	n/a	36.82467651
355	Dairy C	n/a	36.63044357
356	Dairy C	n/a	34.81142934
357	Dairy C	n/a	35.71499634
358	Dairy C	n/a	34.61732737
359	Dairy C	n/a	36.32000923
360	Dairy C	n/a	Undetected
